# Genomic Characteristics of *Desulfonema ishimotonii* Tokyo 01^T^ Implying Horizontal Gene Transfer Among Phylogenetically Dispersed Filamentous Gliding Bacteria

**DOI:** 10.3389/fmicb.2019.00227

**Published:** 2019-02-19

**Authors:** Miho Watanabe, Hisaya Kojima, Kazuhiro Umezawa, Manabu Fukui

**Affiliations:** ^1^Institute of Low Temperature Science, Hokkaido University, Sapporo, Japan; ^2^Japan Society for the Promotion of Science, Tokyo, Japan

**Keywords:** complete genome sequence, genetic transfer, filamentous sulfate-reducing bacterium, *Desulfonema*, genome analysis

## Abstract

*Desulfonema ishimotonii* strain Tokyo 01^T^ is a filamentous sulfate-reducing bacterium isolated from a marine sediment. In this study, the genome of this strain was sequenced and analyzed with a focus on gene transfer from phylogenetically distant organisms. While the strain belongs to the class *Deltaproteobacteria*, hundreds of proteins encoded in the genome showed the highest sequence similarities to those of organisms outside of the class *Deltaproteobacteria*, suggesting that more than 20% of the genome is putatively of foreign origins. Many of these proteins had the highest sequence identities with proteins encoded in the genomes of filamentous bacteria, including giant sulfur oxidizers of the orders *Thiotrichales*, cyanobacteria of various genera, and uncultured bacteria of the candidate phylum KSB3. As mobile genetic elements transferred from phylogenetically distant organisms, putative inteins were identified in the GyrB and DnaE proteins encoded in the genome of strain Tokyo 01^T^. Genes involved in DNA recombination and repair were enriched in comparison to the closest relatives in the same family. Some of these genes were also related to those of organisms outside of the class *Deltaproteobacteria*, suggesting that they were acquired by horizontal gene transfer from diverse bacteria. The genomic data suggested significant genetic transfer among filamentous gliding bacteria in phylogenetically dispersed lineages including filamentous sulfate reducers. This study provides insights into the genomic evolution of filamentous bacteria belonging to diverse lineages, characterized by various physiological functions and different ecological roles.

## Introduction

The genus *Desulfonema* belongs to the family *Desulfobacteraceae* in the class *Deltaproteobacteria* (Kuever, 2014). This genus encompasses mesophilic filamentous sulfate-reducing bacteria which have gliding motility (Kuever et al., 2015). At present, the genus consists of three marine species: *D. limicola* (Widdel et al., 1983), *D. magnum* (Widdel et al., 1983), and *D. ishimotonii* (Fukui et al., 1999). Previous studies have reported that *Desulfonema* species attach to filamentous giant sulfur oxidizers which form conspicuous mats on the sea floor (Fukui et al., 1999; Teske et al., 2009). In a hypersaline lake, *Desulfonema* organisms were abundant in the oxic layer of cyanobacterial mats (Teske et al., 1998; Minz et al., 1999). These findings of physical association imply that *Desulfonema* species have close relationships with filamentous sulfur oxidizers and cyanobacteria, which may drive cycles of sulfur and carbon.

The type strain of *D. ishimotonii*, strain Tokyo 01^T^, was isolated from sulfidic marine sediment of Tokyo Bay, Japan. The 16S rRNA gene sequence analysis demonstrated that strain Tokyo 01^T^ showed 89–90% of sequence similarity to the type strains of *D. limicola* and *D. magnum*. Utilization of alcohols is the distinctive characteristic of *D. ishimotonii*, which has not been found in other *Desulfonema* species (Widdel et al., 1983; Fukui et al., 1999). Here, we describe the complete genome sequence of *Desulfonema ishimotonii* strain Tokyo 01^T^. The sequenced genome was analyzed with a focus on gene transfer among phylogenetically distant lineages.

## Materials and Methods

### DNA Preparation, Sequencing, and Assembly

*Desulfonema ishimotonii* strain Tokyo 01^T^ (=DSM 9680^T^) was grown in artificial seawater medium containing aluminum salt, as described previously (Fukui et al., 1999). Genomic DNA was extracted from the collected cells using a Wizard^®^ Genomic DNA Purification kit (Promega).

Sequencing and library construction were carried out using the PacBio RS II platform. In total, 84,039 polymerase reads (659,435,372 bp) were generated and filtered. The resulting sequences (655,821,304 bp) were assembled into nine linear contigs using FALCON version 0.4.0 (Chin et al., 2016). For the longest contig (6,610,565 bp), a specific PCR primer set was designed by Primer-BLAST (Ye et al., 2012) to bridge both ends of the linear contig. With the primer pair, a PCR product with a length of 1,799 bp was obtained and sequenced. The resulting sequence completely matched that of the contig at both ends, and a circular contig representing a chromosome was constructed by aligning them. The other eight short contigs (max length = 6,714) were directly subjected to further analysis.

### Genome Annotation and Functional Analysis

In the obtained genome, genes were predicted by the MiGAP pipeline (Sugawara et al., 2009). In this pipeline, open-reading frames likely to encode proteins were identified as coding sequences (CDSs) by MetaGene Annotator (Noguchi et al., 2008). In addition, RNAmmer (Lagesen et al., 2007) and tRNAscan-SE (Lowe et al., 1997) were used to identify genes for rRNA and tRNA, respectively. Based on the results of automatic gene prediction by the pipeline, further manual annotation was performed using IMC-GE software (*In Silico* Biology, Yokohama, Japan), as described below. Putative CDSs possessing BLASTP matches with more than 60% coverage, 30% identity, and *E*-values less than e^-8^ were considered as functional genes. The CDSs were annotated as hypothetical proteins when these standard values were not satisfied or the function of the hit was unidentified. Transcription start sites were corrected based on multiple sequence alignments. The eight linear contigs were excluded from further genomic analysis because there was no solid evidence to indicate that they are constituents of the genome of the strain Tokyo 01^T^. CRISPR loci were distinguished using the CRISPR Recognition Tool (Bland et al., 2007). The chromosome was also analyzed with CheckM version 1.0.8 (Parks et al., 2015), to assess its genome completeness and possible contamination in it.

To evaluate the phylogenetic affiliation of each protein-coding gene, all putative protein sequences were searched against the NCBI-nr database (released in 18 February 2018). The top hits with *E*-values less than e^-5^ were regarded as significant hits by BLASTP analysis. The protein-coding genes in the genome sequence were also subjected to analysis on the WebMGA (Wu et al., 2011) for COG annotations. In addition, sequence analysis was carried out using REBASE (Roberts et al., 2015) to identify the DNA restriction and modification system in the genome. Functional domains within proteins were identified by using Pfam protein families database (El-Gebali et al., 2019). For a comparative analysis, COGs annotations of four strains from the genera *Desulfosarcina* and *Desulfococcus* were retrieved from the IMG/M database (Markowitz et al., 2013). These strains were selected as the closest relatives of strain Tokyo 01^T^, on the basis of the 16S rRNA gene sequence similarities higher than 89% (of 1,390 total nucleotide positions).

### Phylogenetic Tree Construction

Phylogenetic analyses were performed for several proteins and inteins encoded in the genome based on their amino acid sequences. They were searched against the public databases (GenBank/EMBL/DDBJ) and aligned with reference sequences using the program CLUSTAL X version 2.1 (Larkin et al., 2007). Selection of the best nucleotide substitution models and construction of the phylogenetic trees were performed using the program MEGA version 7.0.20 (Kumar et al., 2016). All positions with gaps in the alignments were excluded from the calculation.

## Results and Discussion

### General Genomic Features

The obtained genome was comprised of a single 6,610,564 bp circular chromosome (Supplementary Figure S1) and 8 short contigs (total length = 28,713 bp) (Table 1). The complete genome sequence of *D. ishimotonii* Tokyo 01^T^ (=DSM 9680) has been deposited at DDBJ/EMBL/GenBank under the accession number of BEXT01000000.

**Table 1:** General genomic features of *Desulfonema ishimotonii* strain Tokyo 01^T^.

Attributes	Value
Genome size (bp)	6,638,737
Contig	9
DNA-coding region (bp)	5,236,659
DNA G + C content (bp)	3,551,769
Total genes	5,197
RNA count	88
rRNA	12
tRNA	76
Protein-coding genes	5,109
Genes with function prediction	3,249
Genes assigned to COGs	3,616
CRISPR repeats	6

**FIGURE 1:**
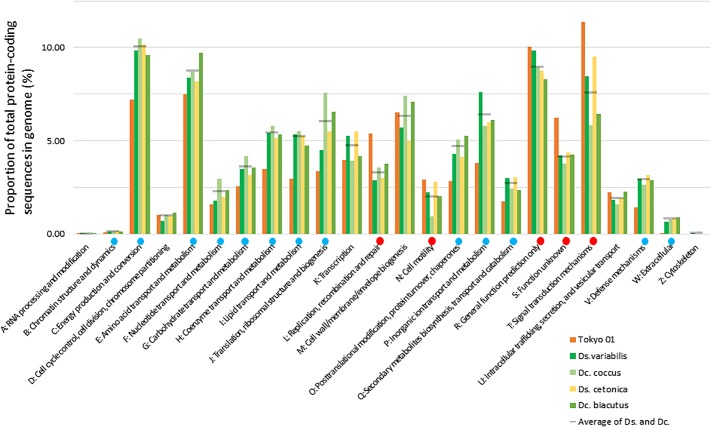
Relative abundances of COG categories in the genomes of strain Tokyo 01^T^ and its closest relatives in the genera *Desulfosarcina* (Ds.) and *Desulfococcus* (Dc.). Gray lines indicate average of the *Desulfosarcina* and *Desulfococcus* species included in the analysis. Red or blue circles indicate that the marked category is significantly enriched or depleted in Tokyo 01’s genome compared to the other genomes, as calculated by the method previously described (Sekiguchi et al., 2015).

Overall, 78.8% of the genome sequence was DNA-coding, with a G + C content of 53.5%. In total, 76 tRNA genes and 12 rRNA genes (four copies each of the 16S, 23S, and 5S rRNA genes) were predicted in the genome. The four copies of the 16S rRNA gene had almost identical sequences, which showed the highest sequence similarities to *Desulfosarcina* and *Desulfococcus* species, rather than the other species in the genus *Desulfonema.* The highest similarity was observed with *Desulfococcus multivorans* DSM 2059^T^ (89.8% in 1,440 nucleotide positions), followed by *Desulfosarcina variabilis* DSM 7267^T^ (89.2% in 1,440 positions). The sequence similarity to the type strains of *D. magnum* and *D. limicola* was 89.0 and 88.6%, respectively (1,394 positions). Approximately 70.3% of proteins encoded in the genome of strain Tokyo 01^T^ were assigned to 20 COG functional categories, and their relative abundances are shown in Figure 1. The completeness of the chromosome as estimated by CheckM was 99.68%, and no contamination was detected using the same tool. The GC skew was unstable throughout the chromosome (Supplementary Figure S1), while that of the *D. multivorans* genome showed the typical pattern, with clear separation at the putative replication origin (Dörries et al., 2016). The atypical pattern of GC skewing was also reported in the genomes of filamentous sulfur oxidizers, *Thioploca ingrica* (Kojima et al., 2015) and *Beggiatoa leptomitoformis* (Orlova et al., 2018).

### Genetic Basis for Carbon Metabolism and Oxygen Tolerance

A previous study reported that the strain oxidizes organic substrates to CO_2_ via the anaerobic C_1_ pathway (Fukui et al., 1999). The strain Tokyo 01^T^ possesses the genes encoding CO dehydrogenase/CO-methylating acetyl-CoA synthase complex (locus tags: DENIS_4589–4592), which is the key enzyme in this pathway. For alcohol oxidation, the genome of the strain harbors five genes encoding alcohol dehydrogenases (DENIS_0326, 0442, 0444, 1430, and 2670). The protein predicted for one of these ORFs (locus tag DENIS_1430) was most similar in amino acid sequence to predicted alcohol dehydrogenases from filamentous or chain-forming sulfur oxidizers, *Beggiatoa* sp. 4572_84 (68%), “*Candidatus* (*Ca*.) Thiomargarita nelsonii” (66%) and *T. ingrica* (65%) (Kojima et al., 2015; Flood et al., 2016; Dombrowski et al., 2017). The other alcohol dehydrogenases predicted from the genome are phylogenetically related to those of the family *Desulfobacteraceae*, consistent with the 16S rRNA gene phylogeny.

The ability to tolerate and/or respire oxygen has been detected in various sulfate-reducing bacteria, including members of the family *Desulfobacteraceae* (Hewitt and Morris, 1975; Hatchikian et al., 1977; Dilling and Cypionka, 1990; Lemos et al., 2001; Kjeldsen et al., 2005). The closest relative of strain Tokyo 01^T^, *D. multivorans* DSM 2059^T^, also possesses genes for oxygen detoxifying enzymes (Dörries et al., 2016). Although strain Tokyo 01^T^ is a strict anaerobe isolated from a sulfidic marine sediment, putative genes for proteins required for oxygen tolerance systems were identified in its genome, including the genes encoding cytochrome *c* oxidase (DENIS_0725, 0727, 0728), cytochrome ubiquinol oxidase (DENIS_3441, 3442, 3677, 3678), superoxide dismutase (DENIS_3256, 5148), and catalase (DENIS_4322). These defense systems against oxidative stress may also be used by *Desulfonema* organisms inhabiting oxygenic environments (Teske et al., 1998; Minz et al., 1999). Despite their close phylogenetic relatedness, the predicted catalase encoded in the genome of strain Tokyo 01^T^ is distinct from that of *D. multivorans* DSM 2069^T^, with an amino acid sequence similarity of 58%. The putative catalase showed the highest sequence similarity (80%) to that of “*Ca.* Kuenenia stuttgartiensis,” a bacterium belonging to the phylum *Planctomycetes* (Parks et al., 2017; Frank et al., 2018). These results suggest that the catalase gene was acquired from a foreign lineage by horizontal gene transfer.

### Phylogenetic Affiliation of Protein-Coding Genes

In addition to the cases of putative alcohol dehydrogenase and catalase mentioned above, many proteins encoded in the genome of strain Tokyo 01^T^ showed the highest sequence similarities to organisms outside the class *Deltaproteobacteria* (Table 2). These results suggest that more than 20% of genes in this genome are putatively of foreign origin.

**Table 2:** Distribution of phylogenetic affiliations of protein-coding genes in circular chromosome of strain Tokyo 01^T^.

Taxon	Number of genes	% of total
*Proteobacteria*	4,222	83.0
*Deltaproteobacteria*	3,933	77.3
*Gammaproteobacteria*	222	4.4
*Alphaproteobacteria*	32	0.6
*Betaproteobacteria*	32	0.6
*Epsilonproteobacteria*	3	0.1
*Cyanobacteria*	94	1.8
*Nitrospirae*	63	1.2
*Firmicutes*	54	1.1
Modulibacteria (KSB3)	54	1.1
*Planctomycetes*	48	0.9
*Chloroflexi*	46	0.9
*Bacteroidetes*	38	0.7
*Spirochaetes*	38	0.7
*Acidobacteria*	11	0.2
*Lentisphaerae*	11	0.2
*Actinobacteria*	10	0.2
*Ignavibacteriae*	10	0.2
Other bacterial phyla	112	2.2
*Archaea*	45	0.9
No hit	231	4.5
Total	5,087	100

*The column of taxon showed the phylogenetic affiliation of organism with the best BLASTP hit against each protein-coding gene.*

In total, 222 proteins (approx. 4.4% of total CDSs) encoded in the genome of strain Tokyo 01^T^ had the best BLASTP hits to gammaproteobacterial proteins, including 55 and 29 proteins from the orders *Thiotrichales* and *Chromatiales*, respectively (Table 3 and Supplementary Table S1). The majority of proteins of these orders are encoded in the genomes of sulfur-oxidizing bacteria (Supplementary Table S1). In particular, those of *Thiotrichales* were mainly identified in the genomes of filamentous and/or giant sulfur oxidizers, including the genera *Beggiatoa*, *Thioploca*, *Thiomargarita*, *Thiothrix*, and “*Ca.* Marithrix.” Most of these proteins with the highest similarities to sulfur-oxidizing bacteria were annotated as hypothetical proteins, and few were identified as functional proteins such as chemotaxis protein CheB, alcohol dehydrogenase, and DNA modification methylase (Supplementary Table S2).

**Table 3:** Order-level classification of phylogenetic distributions of protein-coding genes with the greatest identity to the class *Gammaproteobacteria*.

Order	Number of genes
*Thiotrichales*	55
*Chromatiales*	29
Unclassified (family Competibacteraceae)	21
*Enterobacteraceae*	19
*Alteromonadales*	18
*Methylomonadales*	18
*Pseudomonadales*	13
*Vibrionales*	12
*Oceanospirillales*	10
Other orders (below 10 hits)	27

As shown in Table 2, genes potentially transferred from cyanobacteria comprise 1.8% (94 genes) of the protein-coding genes in the genome of Tokyo 01^T^. Genus-level classification indicated that *Leptolyngbya* may be a major genetic donor in this lineage. Members of the genus *Leptolyngbya* are known as gliding filamentous bacteria, which form clusters or mats (Anagnostidis and Komárek, 1988; Komárek and Anagnostidis, 2005). In addition to this genus, many genera identified in this analysis are known to have filamentous morphology (Table 4). The total count of filamentous genera accounted for half of the best hits against cyanobacteria. The functions of these genes with cyanobacterial origins are still unknown, but some were identified as genes encoding transposases (Supplementary Table S3).

**Table 4:** Genus-level classification of phylogenetic distributions of protein-coding genes with the greatest identity to cyanobacteria.

Genus	Morphology	Number of genes
*Leptolyngbya*	f	15
*Microcystis*	s, a	11
*Anabaena*	f	6
*Oscillatoria*	f	6
*Chroococcidiopsis*	s, a	5
*Acaryochloris*	s	4
*Cyanothece*	v	4
*Nostoc*	f	4
*Calothrix*	f	3
*Pseudanabaena*	f	3
*Chamaesiphon*	s, a	2
*Chlorogloeopsis*	s, a	2
*Coleofasciculus*	f	2
*Moorea*	f	2
*Phormidesmis*	f	2
*Scytonema*	f	2
*Tolypothrix*	f	2
Other genera (1 hits)	–	19
Total	–	94

*Abbreviations: f, filamentous; s, spherical; a, aggregation; v, variable.*

The cases of the order *Thiotrichales* and cyanobacteria indicate that the genome of Tokyo 01^T^ harbors many genes that may have been transferred from filamentous bacteria of phylogenetically distant lineages. In addition, putative genetic donors with filamentous morphology were also found in another lineage without cultivated representatives, known as the candidate phylum KSB3. Among the proteins encoded in the genome of strain Tokyo 01^T^, 100 proteins (2.0% of total) showed the best BLASTP hits to proteins of uncultured bacteria belonging to candidate phyla. Over half of them were potentially transferred from the candidate phylum KSB3 also known as the phylum “Modulibacteria” (Table 2). Among the 54 best hits against KSB3, 29 belonged to “*Ca*. Vecturithrix glanuli,” and 14 showed the greatest identity to “*Ca.* Moduliflexus floccans” (Sekiguchi et al., 2015). Both of these bacteria are known to exhibit filamentous morphology and gliding motility (Sekiguchi et al., 2015). The bacteria of KSB3 are known as the predominant filamentous bacteria in bulking sludge of anaerobic digestion systems (Tanner et al., 2000; Yamada et al., 2007). KSB3 organisms have also been detected as minor members of microbial mats in natural habitats (Wong et al., 2015), but their ecology in the environments remains unknown. In contrast to the genes putatively transferred from the order *Thiotrichales* and cyanobacteria, the genes putatively transferred from KSB3 encode proteins with known functions in most cases. The majority of these genes were predicted as functional genes encoding signal transduction, transporter, or transcriptional proteins (Supplementary Table S4).

The phylogenetic positions of the protein-coding genes showed that the genome of strain Tokyo 01^T^ appears to have been affected by genetic transfer from the order *Thiotrichales*, cyanobacteria, and candidate phylum KSB3. Interestingly, gene sharing between giant sulfur oxidizers and cyanobacteria has repeatedly been reported in previous studies (Mußmann et al., 2007; MacGregor et al., 2013; Flood et al., 2014, 2016; Winkel et al., 2016). As mentioned in the section “Introduction,” organisms from both these groups have been shown to have ecological relationships with filamentous sulfate reducers of the genus *Desulfonema* in the same habitats (Teske et al., 1998; Fukui et al., 1999; Minz et al., 1999; Teske et al., 2009).

### Genetic Elements Relevant to Horizontal Transfer

As described above, many protein-coding genes in the genome of strain Tokyo 01^T^ showed high similarities to those of giant sulfur oxidizers and cyanobacteria. In previous studies, genetic sharing between giant sulfur oxidizers and cyanobacteria was suggested by presence of potential mobile elements and horizontally transferred genes in their genomes (Mußmann et al., 2007; MacGregor et al., 2013; Flood et al., 2014). Further genome analysis of strain Tokyo 01^T^ was performed with a specific focus on these genetic elements investigated in the previous studies as described below.

**FIGURE 2:**
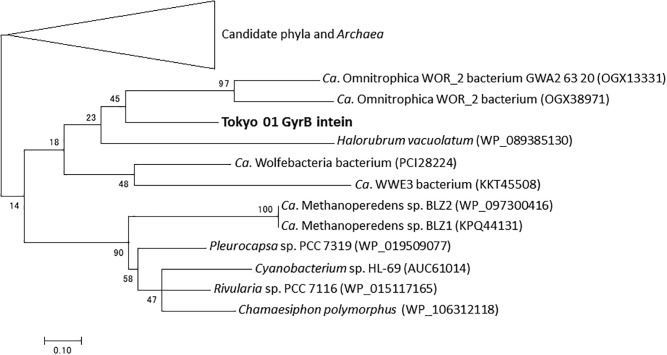
Phylogenetic tree of putative GyrB intein of strain Tokyo 01^T^ and related sequences identified as the top 50 BLAST hits. This tree was constructed by using maximum-likelihood method with LG + G + I model. There were 114 positions in the final dataset. Bootstrap values (percentages of 1,000 replications) are shown at nodes. Neighbor-joining tree are present for comparison in Supplementary Figure S1 in the Supplementary Material.

The DNA gyrase subunit B (GyrB) is a type II topoisomerase indispensable for DNA replication in bacteria (Khodursky et al., 2000). In the genome of strain Tokyo 01^T^, the predicted *gyrB* gene (locus tag DENIS_4377) is located apart from the gene encoding the other subunit of the topoisomerase (*gyrA* gene, locus tag DENIS_5021), as in the genome of *D. multivorans* DSM 2059^T^. GyrB possesses a putative mini-intein which consists of a protein splicing domain but lacks the endonucleolytic domain. The intein constitutes 123 amino acid residues of the 1,042 amino acids in the entire GyrB sequence. A phylogenetic tree of the putative GyrB intein showed that it has a phylogenetic relationship with those found in several candidate phyla, *Archaea*, and *Cyanobacteria* (Figure 2).

**FIGURE 3:**
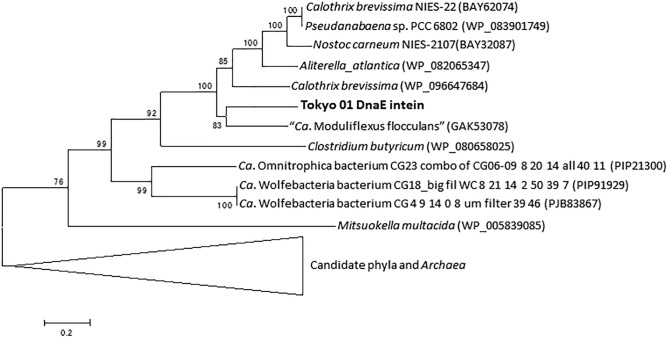
Phylogenetic tree of putative DnaE intein of strain Tokyo 01^T^ and related sequences identified as the top 50 BLAST hits. This tree was constructed by using maximum-likelihood method with LG + G + I model. There were 284 positions in the final dataset. Bootstrap values (percentages of 1,000 replications) are shown at nodes. Neighbor-joining tree are present for comparison in Supplementary Figure S2 in the Supplementary Material.

*dnaE* is the gene for the catalytic subunit of DNA polymerase III subunit alpha (Welch and McHenry, 1982). The putative DnaE protein is encoded by a split gene, with the locus tags DENIS_0455 and DENIS_0456; these two CDSs are adjacent on the genome but are each in different reading frames. A possible intein with a splicing domain was also found in the DnaE protein in the region encoded by DENIS_0456. This intein sequence comprised 415 amino acid residues of the 1,156 amino acids encoded by DENIS_0456, which represents the major part of the DnaE protein. The phylogenetic tree of the putative DnaE intein demonstrated that the intein is related to that of the candidate phylum KSB3 and *Cyanobacteria* (Figure 3). A separate DnaE protein divided by the split inteins was reported in the cyanobacterial lineage (Wu et al., 1998; Caspi et al., 2003), but this was not the case for the strain Tokyo 01^T^.

As shown in Figure 2, 3, the inteins of strain Tokyo 01^T^ in GyrB and DnaE appear to be most similar to possible inteins of several candidate phyla, *Archaea*, and *Cyanobacteria*. With the exceptions of the region corresponding to these inteins, the amino acid sequences of GyrB and DnaE proteins of strain Tokyo 01^T^ are closely related to those of the *Desulfobacteraceae* species. The inteins in GyrB and DnaE have not been found in the closest relatives of strain Tokyo 01^T^. The GyrB and DnaE inteins were found in the genomes of *Beggiatoaceae* (MacGregor et al., 2013; Flood et al., 2016) which may be a major donors of foreign genes to strain Tokyo 01^T^. Nevertheless, their intein sequences were not closely related to that of strain Tokyo 01^T^.

As another genetic element of foreign origin, introns in the 23S rRNA gene were identified in the genomes of *Beggiatoaceae* organisms and cyanobacteria (MacGregor et al., 2013). In the 23S rRNA gene of strain Tokyo 01^T^, however, no intron was identified. XisH and XisI are proteins involved in heterocyst differentiation of some cyanobacteria (Carrasco et al., 2005; Henson et al., 2008), and genes encoding these proteins have been identified in the genomes of giant filamentous sulfur oxidizers (Mußmann et al., 2007; MacGregor et al., 2013). The genes encoding proteins related to XisH or XisI were not found in the genome of strain Tokyo 01^T^.

### DNA Replication, Recombination, and Repair Systems

To reveal the genomic feature of strain Tokyo 01^T^, comparative analysis against the closest relatives was performed on the basis of the relative abundance of CDSs classified into COG categories (Figure 1). This analysis revealed that the genome of Tokyo 01^T^ harbors a high proportion of genes involved in DNA recombination and repair (L), cell motility (N), and signal transduction (T). Consequently, genes of other categories were generally depleted in comparison to the closest relatives. Among the enriched categories, COG category L was the most highly enriched in the genome of strain Tokyo 01^T^. The genes in this category may also be related to genetic transfer from giant sulfur oxidizers and cyanobacteria. More detailed properties of the genes for replication, recombination, and repair systems in the genome of strain Tokyo 01^T^ are described below.

The DNA repair systems protect bacterial cells against genetic damage. The nucleotide excision repair (NER) system is one of the most important mechanisms for DNA damage removal and is widely conserved in bacteria. The complete gene set for the NER systems has also been found in some euryarchaeota, whereas archaea of other lineages may have other unknown systems (Grogan, 2000; Costa et al., 2003). In the genome of strain Tokyo 01^T^, *uvrA*, *uvrB*, *uvrC*, and *uvrD* were identified as the NER genes (Supplementary Table S5). There were two copies of predicted *uvrB* and *uvrD* genes with different sequences. One of the putative *uvrD* genes (DENIS_2690) encoded a protein more closely related to that of archaea, rather than deltaproteobacteria. Previous studies have demonstrated that the phylogeny of the NER system genes exhibit significant incongruence as a result of horizontal gene transfers (Denamur et al., 2000; Martins-Pinheiro et al., 2004). However, phylogenetic positions of the other NER genes seemed to be generally consistent with the 16S rRNA gene phylogeny.

**FIGURE 4:**
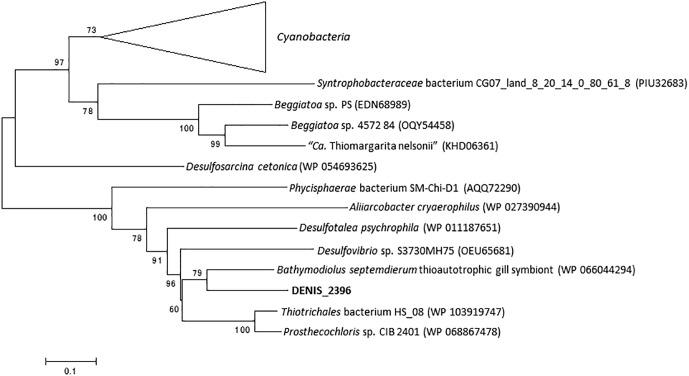
RecQ phylogeny of the strain Tokyo 01^T^ and *Desulfosarcina cetonica*. The top 55 BLAST hits to DENIS_2396 were used as the dataset to construct the tree. There were 662 positions in the final dataset. This tree was constructed by using maximum-likelihood method with LG + G + I model. Bootstrap values (percentages of 1,000 replications) are shown at nodes.

**FIGURE 5:**
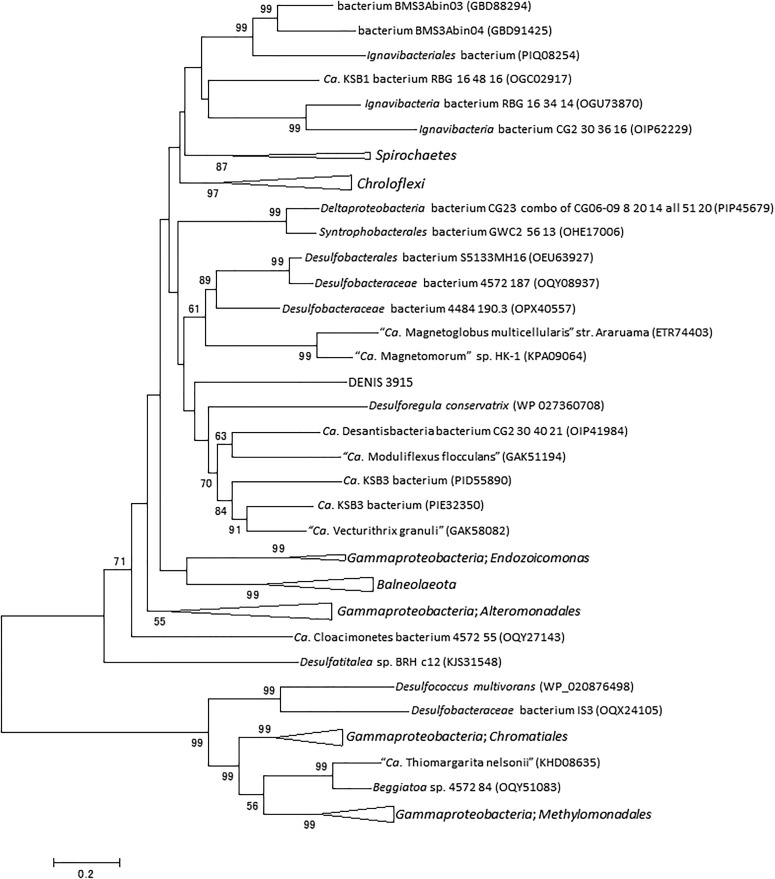
RecQ phylogeny of the strain Tokyo 01^T^ and *D. multivorans*. The dataset consists the top 50 BLAST hits to DENIS_3915 and 20 hits to the RecQ of *D. multivorans*. There were 560 positions in the final dataset. This tree was constructed by using maximum-likelihood method with LG + G + I model. Bootstrap values (percentages of 1,000 replications) lower than 50% are shown at nodes.

The RecQ family DNA helicases play an important role in genome maintenance and are ubiquitously conserved in life forms ranging from bacteria to eukaryotes (Nakayama et al., 1984; Kusano et al., 1994; Chu and Hickson, 2009). In general, bacterial genomes encode only one RecQ homolog (Kaiser et al., 2017), although exceptions can be seen in genomes of *Staphylococcus aureus* subsp. *aureus* DR10 (accession number: AIDT01000000) and *S. aureus* subsp. *aureus* 71193 (CP003045). In the genome of strain Tokyo 01^T^, two genes encoding RecQ were identified (DENIS_2369 and DENIS_3915). The proteins encoded by these predicted genes are phylogenetically distant from each other. The protein encoded by DENIS_2369 protein had the highest amino acid sequence similarity (78%) with RecQ of *Bathymodiolus septemdierum*, a gammaproteobacterial thioautotrophic gill symbiont (Ikuta et al., 2016). The closer relationship with gammaproteobacterial proteins was confirmed by constructing a phylogenetic tree (Figure 4). The other putative RecQ, encoded by DENIS_3915, had the highest sequence similarity with the RecQ protein of candidate phylum “Desantisbacteria” bacterium CG2_30_40_21 (Probst et al., 2017), with a similarity of 58%. Phylogenetic analysis showed that this protein is distantly related to the RecQ helicases of some bacteria of the family *Desulfobacteraceae* (Figure 5). While all known functional domains of the RecQ helicase were identified in the protein encoded by DENIS_3915, the protein encoded by DENIS_2369 seemed to possess only the ATPase domain, which is the most well-conserved element among three RecQ-characteristic domains (Kaiser et al., 2017).

A search against the REBASE database identified 40 genes for restriction-modification systems in the genome of strain Tokyo 01^T^ (Supplementary Table S6). The proteins encoded by these predicted genes showed the highest sequence similarities to proteins of prokaryotes belonging to diverse taxa, including *Gammaproteobacteria* (8 out of 40) and *Cyanobacteria* (5), as major lineages outside of *Deltaproteobacteria*. Despite their crucial role in defense against foreign DNA, previous studies have demonstrated that the genes for restriction–modification systems have undergone frequent genetic exchange between species (Kita et al., 1999; Kobayashi et al., 1999; Rocha et al., 1999; Nobusato et al., 2000) and quick evolution (Lauster, 1989; Jeltsch and Pingoud, 1996). It was also shown that these genes in “*Ca.* Maribeggiatoa” are related to a phylogenetically wide range of organisms, suggesting their foreign origins (MacGregor et al., 2013).

Duplications of the genes for DNA repair system possibly improve response to environmental stress (Van Sluys et al., 2002; Martins-Pinheiro et al., 2004). The multiple genes for RecQ helicases and other DNA repair genes in strain Tokyo 01^T^ may function to maintain genomic stability during aggressive horizontal genetic transfer and facilitate response to environmental changes.

## Conclusion

In this study, we showed that the genome of strain Tokyo 01^T^ appears to have many protein-coding genes with wide phylogenetic affiliations, implying extensive genetic transfer from diverse organisms. A considerable number of genetic elements were inferred to be transferred from filamentous bacteria, including gammaproteobacterial sulfur oxidizers, cyanobacteria, and organisms of the candidate phylum KSB3. The enhanced DNA repair systems of strain Tokyo 01^T^ might have helped genomic maintenance during horizontal gene transfer. We conclude that the strain Tokyo 01^T^ obtained genetic elements from gliding filamentous bacteria belonging to various lineages. The acquired foreign genes might have facilitated acquisition of new niches (Lawrence, 1999). This study highlights the unexplored aspect of genomic evolutions of filamentous bacteria, which may be intertwined with organisms of different physiological functions and ecological roles.

## Author Contributions

MF and HK supervised the study. HK performed the cultivation and genomic DNA preparation for the analysis. KU carried out data analysis to obtain the genome sequence. MW designed the study and carried out bioinformatics analysis based on the obtained genome. MW and HK wrote the manuscript. All authors discussed the data and approved the final manuscript.

## Conflict of Interest Statement

The authors declare that the research was conducted in the absence of any commercial or financial relationships that could be construed as a potential conflict of interest.

## Supplementary Material

FIGURE S1 Circular map of the single chromosome of strain Tokyo 01^T^. From outside to the center; genes on forward strand, genes on reverse strand, G + C content, GC skew. Positive or negative values in G + C content and GC skew were shown as pink or green, respectively.

FIGURE S2 Neighbor-joining tree of putative GyrB intein of strain Tokyo 01^T^ and other organisms.

FIGURE S3 Neighbor-joining tree of putative DnaE intein of strain Tokyo 01^T^ and other organisms.
