# Supplementary figures and images for: Genomic Characteristics of Desulfonema ishimotonii Tokyo 01T Implying Horizontal Gene Transfer Among Phylogenetically Dispersed Filamentous Gliding Bacteria
Source: Front Microbiol. 2019 Feb 19;10:227. doi: 10.3389/fmicb.2019.00227 (PMC6390638; doi:10.3389/fmicb.2019.00227)

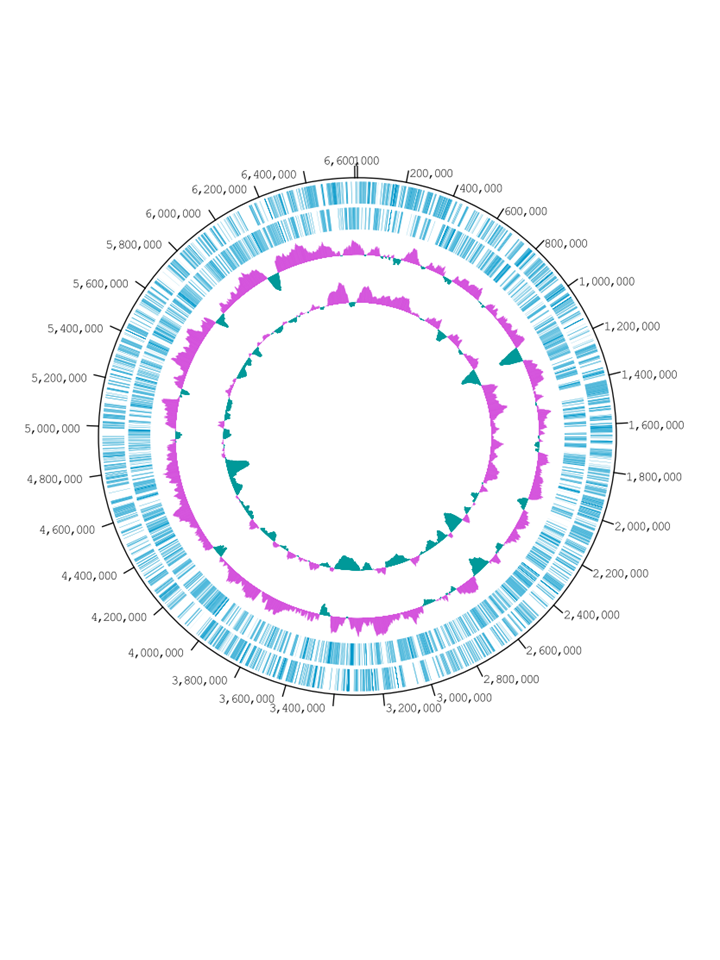

Supplement: FIGURE S1 — Circular map of the single chromosome of strain Tokyo 01T. From outside to the center; genes on forward strand, genes on reverse strand, G + C content, GC skew. Positive or negative values in G + C content and GC skew were shown as pink or green, respectively. [file Image_1.TIF]

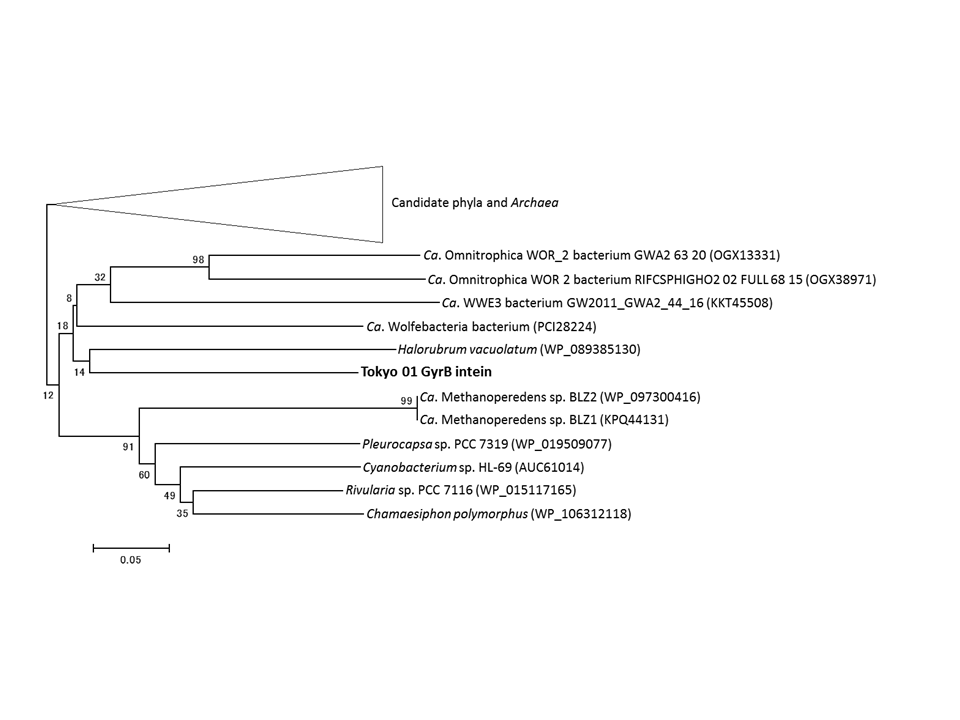

Supplement: FIGURE S2 — Neighbor-joining tree of putative GyrB intein of strain Tokyo 01T and other organisms. [file Image_2.TIF]

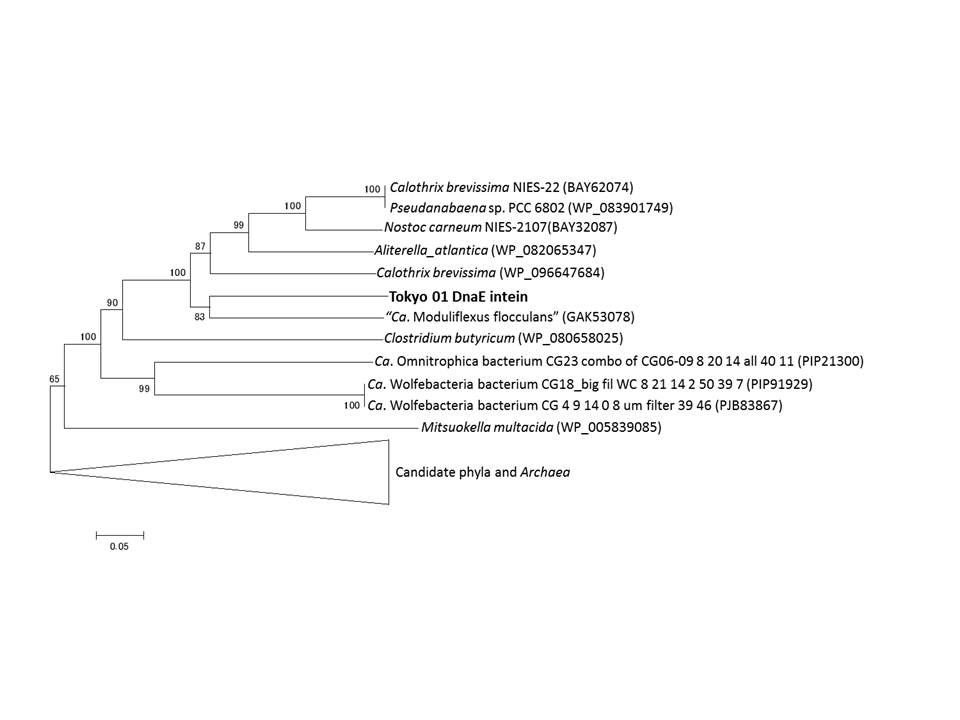

Supplement: FIGURE S3 — Neighbor-joining tree of putative DnaE intein of strain Tokyo 01T and other organisms. [file Image_3.TIF]
