# Supplementary material for: Genomic Characteristics of Desulfonema ishimotonii Tokyo 01T Implying Horizontal Gene Transfer Among Phylogenetically Dispersed Filamentous Gliding Bacteria
Source: Front Microbiol. 2019 Feb 19;10:227. doi: 10.3389/fmicb.2019.00227 (PMC6390638; doi:10.3389/fmicb.2019.00227)
Supplement: Supplementary file 4 [file Table_1.DOCX]

**Table S1**. Number of CDSs with the top hit of the class *Gammaproteobacteria.*

| Taxon | Hits | Breakdown | Hits |
| --- | --- | --- | --- |
| Order *Thiotrichales* | 55 | *Beggiatoa* | 14 |
|  |  | *Ca*. Thiomargarita nelsonii | 9 |
|  |  | *Thiotrichales* | 8 |
|  |  | *Ca*. Marithrix sp. | 7 |
|  |  | *Thioploca* | 7 |
|  |  | *Francisella* | 4 |
|  |  | *Thiothrix* | 2 |
|  |  | *Cycloclasticus* | 1 |
|  |  | *Methylophaga* | 1 |
|  |  | *Thiomicrospira* | 1 |
|  |  | *Thiotrichaceae* | 1 |
|  |  |  |  |
| *Order Chromatiales* | 29 | *Thiorhodovibrio* | 6 |
|  |  | *Thiocapsa* | 3 |
|  |  | *Thiorhodococcus* | 3 |
|  |  | *Ca.* Thiodictyon syntrophicum | 2 |
|  |  | *Ca*. Thiosymbion oneisti | 2 |
|  |  | *Marichromatium* | 2 |
|  |  | *Nitrosococcus* | 2 |
|  |  | *Thioflavicoccus* | 2 |
|  |  | *Acidihalobacter* | 1 |
|  |  | *Halothiobacillus* | 1 |
|  |  | *Imhoffiella* | 1 |
|  |  | *Rheinheimera* | 1 |
|  |  | *Thiohalocapsa* | 1 |
|  |  | *Woeseia* | 1 |
|  |  | uncultivated *Chromatiales* | 1 |
|  |  |  |  |
| unclassified, family ”Competibacteraceae” | 21 | *Ca*. Contendobacter odensis | 18 |
|  |  | *Ca*. Competibacter denitrificans | 3 |
|  |  |  |  |
| Order *Enterobacteriales* | 19 | *Pectobacterium* | 10 |
|  |  | *Escherichia* | 3 |
|  |  | *Dickeya* | 1 |
|  |  | *Enterobacter* | 1 |
|  |  | *Klebsiella* | 1 |
|  |  | *Providencia* | 1 |
|  |  | *Shigella* | 1 |
|  |  | *Xenorhabdus* | 1 |
|  |  |  |  |
| Order *Alteromonadales* | 18 | *Shewanella* | 6 |
|  |  | *Alteromonas* | 2 |
|  |  | *Pseudoalteromonas* | 2 |
|  |  | *Catenovulum* | 1 |
|  |  | *Colwellia* | 1 |
|  |  | *Glaciecola* | 1 |
|  |  | *Mangrovitalea* | 1 |
|  |  | *Marinobacter* | 1 |
|  |  | *Psychromonas* | 1 |
|  |  | uncultivated *Alteromonadaceae* | 2 |
|  |  |  |  |
| Order *Methylomonadales* | 18 | *Methylobacter* | 3 |
|  |  | *Bathymodiolus* gill symbiont | 2 |
|  |  | *Methylocaldum* | 2 |
|  |  | *Methylomonas* | 2 |
|  |  | *Methylovulum* | 2 |
|  |  | *Crenothrix* | 1 |
|  |  | *Methylococcus* | 1 |
|  |  | *Methylomagnum* | 1 |
|  |  | *Methylomarinum* | 1 |
|  |  | *Methyloprofundus* | 1 |
|  |  | *Methyloterricola* | 1 |
|  |  | uncultivated *Methylococcales* | 1 |
|  |  |  |  |
| Order *Pseudomonadales* | 13 | *Pseudomonas* | 9 |
|  |  | *Moraxella* | 3 |
|  |  | *Ventosimonas* | 1 |
|  |  |  |  |
| Order *Vibrionales* | 12 | *Vibrio* | 10 |
|  |  | *Aliivibrio* | 1 |
|  |  | *Thaumasiovibrio* | 1 |
|  |  |  |  |
| Order *Oceanospirillales* | 10 | *Hahella* | 6 |
|  |  | *Endozoicomonas* | 2 |
|  |  | *Bacterioplanes* | 1 |
|  |  | *Nitrincola* | 1 |
|  |  |  |  |
| Other orders (below 10 hits) | 27 | - | - |
