# Supplementary material for: Genomic Characteristics of Desulfonema ishimotonii Tokyo 01T Implying Horizontal Gene Transfer Among Phylogenetically Dispersed Filamentous Gliding Bacteria
Source: Front Microbiol. 2019 Feb 19;10:227. doi: 10.3389/fmicb.2019.00227 (PMC6390638; doi:10.3389/fmicb.2019.00227)
Supplement: Supplementary file 5 [file Table_2.DOCX]

**Table S2**. Strain Tokyo 01^T^’s ORFs with the highest similarity to proteins of gammaproteobacterial sulfur-oxidizers

| Locus tag | Protein name | Organism name | e-value | identity |
| --- | --- | --- | --- | --- |
| DENIS_1799 | chemotaxis protein CheB | *Beggiatoa leptomitiformis* | 5.09E-19 | 51.9 |
| DENIS_2216 | DUF2259 domain-containing protein | *Beggiatoa leptomitiformis* | 1.54E-25 | 29.6 |
| DENIS_1430 | alcohol dehydrogenase | *Beggiatoa* sp. 4572_84 | 1.17E-164 | 67.6 |
| DENIS_3579 | hypothetical protein B6247_18055 | *Beggiatoa* sp. 4572_84 | 3.52E-90 | 36.2 |
| DENIS_4034 | hypothetical protein B6247_06230 | *Beggiatoa* sp. 4572_84 | 0 | 59.8 |
| DENIS_1909 | hypothetical protein BWK79_04210 | *Beggiatoa* sp. IS2 | 5.19E-51 | 69.8 |
| DENIS_4440 | hypothetical protein BWK79_13330 | *Beggiatoa* sp. IS2 | 1.81E-43 | 61.2 |
| DENIS_4456 | hypothetical protein BWK79_13330 | *Beggiatoa* sp. IS2 | 1.9E-46 | 62.4 |
| DENIS_4464 | hypothetical protein BWK79_13330 | *Beggiatoa* sp. IS2 | 1.90E-46 | 62.4 |
| DENIS_0387 | conserved hypothetical protein | *Beggiatoa* sp. PS | 2.01E-67 | 41.9 |
| DENIS_1811 | ISBma1, transposase | *Beggiatoa* sp. PS | 2.19E-13 | 49.3 |
| DENIS_2236 | conserved hypothetical protein | *Beggiatoa* sp. PS | 0 | 76.7 |
| DENIS_3274 | hypothetical protein BGP_2294 | *Beggiatoa* sp. PS | 5.67E-71 | 47.5 |
| DENIS_4093 | Transposase, IS630 | *Beggiatoa* sp. PS | 5.42E-24 | 39.1 |
| DENIS_1055 | DNA invertase | *Ca.* Thiomargarita nelsonii | 5.96E-79 | 57.8 |
| DENIS_1136 | hypothetical protein OT06_33895 | *Ca*. Thiomargarita nelsonii | 8.95E-128 | 54.2 |
| DENIS_2056 | hypothetical protein OT06_41600 | *Ca*. Thiomargarita nelsonii | 0 | 55.2 |
| DENIS_2265 | peptidase | *Ca*. Thiomargarita nelsonii | 1.87E-94 | 63.9 |
| DENIS_2734 | hypothetical protein OT06_25090 | *Ca*. Thiomargarita nelsonii | 6.55E-36 | 47.6 |
| DENIS_3748 | hypothetical protein OT06_54140 | *Ca*. Thiomargarita nelsonii | 1.50E-13 | 35.1 |
| DENIS_4153 | hypothetical protein OT06_60180 | *Ca*. Thiomargarita nelsonii | 4.43E-127 | 43.4 |
| DENIS_4978 | hypothetical protein OT06_59290 | *Ca*. Thiomargarita nelsonii | 5.47E-103 | 56.4 |
| DENIS_5024 | hypothetical protein THIOM_005662 | *Ca*. Thiomargarita nelsonii | 3.81E-83 | 53.1 |
| DENIS_3189 | hypothetical protein BWK78_07910 | T*hiotrichaceae* bacterium IS1 | 2.37E-22 | 28.2 |
| DENIS_1666 | L-glutamyl-decarboxylase | *Thiotrichales* bacterium HS_08 | 9.47E-26 | 55.2 |
| DENIS_1667 | L-glutamyl-decarboxylase | *Thiotrichales* bacterium HS_08 | 7.01E-86 | 47.4 |
| DENIS_1956 | nicotinamidase/pyrazinamidase | *Thiotrichales* bacterium HS_08 | 1.93E-106 | 58.5 |
| DENIS_1957 | Uncharacterised protein | *Thiotrichales* bacterium HS_08 | 1.99E-93 | 59.3 |
| DENIS_3776 | hypothetical protein | *Thiotrichales* bacterium HS_08 | 1.96E-106 | 58.5 |
| DENIS_3777 | VWA domain-containing protein | *Thiotrichales* bacterium HS_08 | 2.03E-93 | 59.3 |
| DENIS_4775 | hypothetical protein | *Thiotrichales* bacterium HS_08 | 4.92E-66 | 43.2 |
| DENIS_0912 | hypothetical protein COB71_13275 | *Thiotrichales* bacterium | 1.51E-38 | 41.5 |
| DENIS_1061 | hypothetical protein | *Ca*. Marithrix sp. Canyon 246 | 8.41E-61 | 55.5 |
| DENIS_1137 | hypothetical protein | *Ca*. Marithrix sp. Canyon 246 | 1.13E-64 | 56.7 |
| DENIS_1690 | hypothetical protein | *Ca*. Marithrix sp. Canyon 246 | 1.79E-162 | 72.3 |
| DENIS_1691 | DNA modification methylase | *Ca*. Marithrix sp. Canyon 246 | 0 | 63.9 |
| DENIS_3029 | DNA adenine methylase | *Ca*. Marithrix sp. Canyon 246 | 2.96E-161 | 71.8 |
| DENIS_4006 | hypothetical protein | *Ca*. Marithrix sp. Canyon 246 | 2.09E-24 | 39.6 |
| DENIS_4046 | hypothetical protein | *Ca*. Marithrix sp. Canyon 246 | 9.48E-14 | 21.9 |
| DENIS_0291 | hypothetical protein | *Thioploca ingrica* | 1.62E-52 | 65.9 |
| DENIS_2352 | HEAT repeat domain-containing protein | *Thioploca ingrica* | 7.94E-28 | 32.4 |
| DENIS_2674 | PAS domain S-box protein | *Thioploca ingrica* | 8.68E-128 | 39.2 |
| DENIS_2723 | lipase family protein | *Thioploca ingrica* | 1.78E-63 | 41.7 |
| DENIS_3100 | type VI secretion system tip protein VgrG | *Thioploca ingrica* | 0 | 51.8 |
| DENIS_4011 | PBS lyase HEAT-like repeat protein | *Thioploca ingrica* | 4.12E-81 | 47.6 |
| DENIS_5168 | hypothetical protein | *Thioploca ingrica* | 1.97E-64 | 52.4 |
| DENIS_1601 | hypothetical protein BWK73_20865 | *Thiothrix lacustris* | 0 | 59.3 |
| DENIS_2406 | type I-E CRISPR-associated protein Cse1/CasA | *Thiothrix lacustris* | 7.73E-10 | 22.7 |
| DENIS_3214 | plasmid maintenance system killer protein | *Thiomicrospira* sp. CG2_30_44_34 | 2.15E-50 | 76.3 |
| DENIS_3189 | hypothetical protein BWK78_07910 | *Thiotrichaceae* bacterium IS1 | 2.37E-22 | 28.2 |
