# Supplementary material for: Genomic Characteristics of Desulfonema ishimotonii Tokyo 01T Implying Horizontal Gene Transfer Among Phylogenetically Dispersed Filamentous Gliding Bacteria
Source: Front Microbiol. 2019 Feb 19;10:227. doi: 10.3389/fmicb.2019.00227 (PMC6390638; doi:10.3389/fmicb.2019.00227)
Supplement: Supplementary file 6 [file Table_3.docx]

**Table S3.** Strain Tokyo 01^T^’s ORFs with the highest similarity to cyanobacterial proteins

| locus tag | Protein name | Organism name | e-value | identity |
| --- | --- | --- | --- | --- |
| DENIS_1356 | ribonuclease HI | *Leptolyngbya ohadii* | 3.84E-77 | 69.9 |
| DENIS_1366 | TIR domain-containing protein | *Leptolyngbya* sp. Heron Island J | 1.3E-125 | 44.2 |
| DENIS_0752 | transposase | *Leptolyngbya* sp. PCC 7375 | 6.12E-71 | 51.5 |
| DENIS_0754 | transposase | *Leptolyngbya* sp. PCC 7375 | 4.46E-43 | 54.0 |
| DENIS_1921 | transposase | *Leptolyngbya* sp. PCC 7375 | 6.12E-71 | 51.5 |
| DENIS_2534 | transposase | *Leptolyngbya* sp. PCC 7375 | 6.22E-71 | 51.5 |
| DENIS_2687 | transposase | *Leptolyngbya* sp. PCC 7375 | 6.22E-71 | 51.5 |
| DENIS_2816 | transposase | *Leptolyngbya* sp. PCC 7375 | 1.13E-70 | 51.5 |
| DENIS_2865 | transposase | *Leptolyngbya* sp. PCC 7375 | 6.22E-71 | 51.5 |
| DENIS_3251 | transposase | *Leptolyngbya* sp. PCC 7375 | 1.13E-70 | 51.5 |
| DENIS_3405 | transposase | *Leptolyngbya* sp. PCC 7375 | 6.22E-71 | 51.5 |
| DENIS_4074 | transposase | *Leptolyngbya* sp. PCC 7375 | 3.47E-66 | 49.5 |
| DENIS_4418 | transposase | *Leptolyngbya* sp. PCC 7375 | 6.22E-71 | 51.5 |
| DENIS_4570 | transposase | *Leptolyngbya* sp. PCC 7375 | 1.13E-70 | 51.5 |
| DENIS_3092 | restriction endonuclease | *Leptolyngbya valderiana* | 3.36E-62 | 50.0 |
| DENIS_0751 | hypothetical protein | *Microcystis aeruginosa* | 4.68E-42 | 49.7 |
| DENIS_2535 | hypothetical protein | *Microcystis aeruginosa* | 1.05E-44 | 50.0 |
| DENIS_2686 | hypothetical protein | *Microcystis aeruginosa* | 1.05E-44 | 50.0 |
| DENIS_2815 | hypothetical protein | *Microcystis aeruginosa* | 1.05E-44 | 50.0 |
| DENIS_2866 | hypothetical protein | *Microcystis aeruginosa* | 1.05E-44 | 50.0 |
| DENIS_3250 | hypothetical protein | *Microcystis aeruginosa* | 1.05E-44 | 50.0 |
| DENIS_3406 | hypothetical protein | *Microcystis aeruginosa* | 1.05E-44 | 50.0 |
| DENIS_3755 | hypothetical protein | *Microcystis aeruginosa* | 4.98E-87 | 53.4 |
| DENIS_4075 | hypothetical protein | *Microcystis aeruginosa* | 1.96E-44 | 51.7 |
| DENIS_4419 | hypothetical protein | *Microcystis aeruginosa* | 1.05E-44 | 50.0 |
| DENIS_4569 | hypothetical protein | *Microcystis aeruginosa* | 1.05E-44 | 50.0 |
| DENIS_3536 | IS1 family transposase | *Anabaena cylindrica* | 1.9E-72 | 75.9 |
| DENIS_3266 | hypothetical protein | *Anabaena* sp. 90 | 1.7E-66 | 52.7 |
| DENIS_4154 | hypothetical protein AN485_13435 | *Anabaena* sp. MDT14b | 1.11E-29 | 36.5 |
| DENIS_3941 | hypothetical protein | *Anabaena* sp. PCC 7108 | 8.31E-12 | 25.7 |
| DENIS_0366 | hypothetical protein | *Anabaena* sp. WA102 | 2.94E-88 | 63.2 |
| DENIS_3533 | hypothetical protein AN488_20660 | *Anabaena* sp. WA113 | 7.13E-13 | 37.5 |
| DENIS_3703 | peptidylprolyl isomerase | cyanobacterium PCC 7702 | 4E-20 | 34.0 |
| DENIS_0920 | hypothetical protein B7486_44445 | cyanobacterium TDX16 | 5.45E-107 | 43.3 |
| DENIS_1960 | hypothetical protein B7486_44445 | cyanobacterium TDX16 | 2.43E-108 | 43.6 |
| DENIS_2114 | hypothetical protein B7486_44445 | cyanobacterium TDX16 | 5.45E-107 | 43.3 |
| DENIS_2298 | hypothetical protein B7486_09090 | cyanobacterium TDX16 | 1.14E-97 | 64.6 |
| DENIS_2681 | hypothetical protein B7486_44445 | cyanobacterium TDX16 | 3.47E-10 | 75.7 |
| DENIS_1755 | sugar ABC transporter permease | *Oscillatoria acuminata* | 4.38E-125 | 65.5 |
| DENIS_1756 | sugar ABC transporter substrate-binding protein | *Oscillatoria acuminata* | 0 | 58.6 |
| DENIS_1368 | alpha-amylase | *Oscillatoria nigroviridis* | 4.53E-99 | 35.8 |
| DENIS_2832 | PAS domain S-box protein | *Oscillatoria nigroviridis* | 7.74E-46 | 35.2 |
| DENIS_3545 | ATP-grasp domain-containing protein | *Oscillatoria nigroviridis* | 9.13E-160 | 59.3 |
| DENIS_4294 | transposase | *Oscillatoria* sp. PCC 10802 | 5.61E-29 | 63.0 |
| DENIS_0936 | hypothetical protein | *Acaryochloris marina* | 5.75E-21 | 48.3 |
| DENIS_1471 | hypothetical protein | *Acaryochloris marina* | 5.65E-28 | 42.0 |
| DENIS_3144 | DUF3592 domain-containing protein | *Acaryochloris marina* | 3.75E-07 | 41.9 |
| DENIS_1925 | hypothetical protein | *Acaryochloris* sp. CCMEE 5410 | 5.6E-35 | 46.4 |
| DENIS_2387 | hypothetical protein PCC7424_4760 | *Cyanothece* sp. PCC 7424 | 9.16E-93 | 41.4 |
| DENIS_2388 | hypothetical protein | *Cyanothece* sp. PCC 7424 | 4.19E-156 | 55.0 |
| DENIS_2685 | hypothetical protein | *Cyanothece* sp. PCC 7425 | 0 | 33.6 |
| DENIS_4498 | B12-binding domain-containing radical SAM protein | *Cyanothece* sp. PCC 8802 | 0 | 60.8 |
| DENIS_2092 | IS630 family transposase | *Nostoc flagelliforme* | 4.55E-11 | 65.0 |
| DENIS_3688 | HNH endonuclease | *Nostoc linckia* | 0 | 64.3 |
| DENIS_4749 | hypothetical protein | *Nostoc linckia* | 3.67E-14 | 61.4 |
| DENIS_1939 | RHS repeat-associated core domain-containing protein | *Nostoc* sp. PCC 7524 | 2.07E-24 | 58.8 |
| DENIS_0696 | guanosine-3',5'-bis(diphosphate) 3'-pyrophosphohydrolase | *Calothrix brevissima* | 5.39E-85 | 65.9 |
| DENIS_2743 | non-ribosomal peptide synthetase | *Calothrix brevissima* | 0 | 45.8 |
| DENIS_3419 | WG repeat-containing protein | *Calothrix* sp. HK-06 | 1.66E-36 | 36.2 |
| DENIS_3544 | MFS transporter | *Pseudanabaena* sp. PCC 7367 | 0 | 38.8 |
| DENIS_4461 | IS982 family transposase | *Pseudanabaena* sp. PCC 7367 | 5.28E-44 | 59.5 |
| DENIS_1132 | hypothetical protein | *Pseudanabaena* sp. SR411 | 6.32E-125 | 49.6 |
| DENIS_1305 | beta-galactosidase/beta-glucuronidase | *Chamaesiphon minutus* | 0 | 49.5 |
| DENIS_2165 | DUF2813 domain-containing protein | *Chamaesiphon minutus* | 0 | 59.7 |
| DENIS_0182 | acyltransferase | *Chlorogloeopsis fritschii* | 2.01E-77 | 39.4 |
| DENIS_3758 | 2-hydroxy-3-keto-5-methylthiopentenyl-1-phosphate phosphatase | *Chlorogloeopsis fritschii* | 7.15E-78 | 54.8 |
| DENIS_2588 | hypothetical protein | *Coleofasciculus chthonoplastes* | 4.4E-62 | 78.2 |
| DENIS_3377 | NACHT domain-containing protein | *Coleofasciculus chthonoplastes* | 0 | 52.9 |
| DENIS_2587 | DNA methyltransferase | *Moorea producens* | 0 | 70.2 |
| DENIS_2589 | hypothetical protein | *Moorea producens* | 1.72E-70 | 73.7 |
| DENIS_2264 | M6 family metalloprotease domain-containing protein | *Oscillatoriales* cyanobacterium MTP1 | 0 | 53.5 |
| DENIS_1407 | hypothetical protein BCD67_04325 | *Oscillatoriales* cyanobacterium USR001 | 4.55E-159 | 56.4 |
| DENIS_1753 | sn-glycerol-3-phosphate ABC transporter ATP-binding protein UgpC | *Phormidesmis priestleyi* | 2.88E-142 | 54.6 |
| DENIS_2308 | hypothetical protein | *Phormidesmis priestleyi* | 2.46E-31 | 52.7 |
| DENIS_3371 | hypothetical protein | *Scytonema* sp. HK-05 | 0 | 54.1 |
| DENIS_3729 | hypothetical protein | *Scytonema tolypothrichoides* | 1.37E-33 | 46.7 |
| DENIS_3372 | CHAT domain-containing protein | *Tolypothrix* sp. NIES-4075 | 1.58E-52 | 59.9 |
| DENIS_4047 | sulfatase-modifying factor protein | *Tolypothrix* sp. NIES-4075 | 1.39E-31 | 24.8 |
