# Supplementary material for: Genomic Characteristics of Desulfonema ishimotonii Tokyo 01T Implying Horizontal Gene Transfer Among Phylogenetically Dispersed Filamentous Gliding Bacteria
Source: Front Microbiol. 2019 Feb 19;10:227. doi: 10.3389/fmicb.2019.00227 (PMC6390638; doi:10.3389/fmicb.2019.00227)
Supplement: Supplementary file 7 [file Table_4.docx]

**Table S4**. Strain Tokyo 01^T^’s ORFs with the highest similarity to proteins of “*Ca*. Modulibacteria” organism

| locus tag | Protein name | Organism name | e-value | identity |
| --- | --- | --- | --- | --- |
| DENIS_0194 | hypothetical protein U14_01102 | *Ca*. Moduliflexus flocculans | 9.26E-18 | 44.9 |
| DENIS_0456 | DNA polymerase III, alpha subunit | *Ca*. Moduliflexus flocculans | 0 | 56.6 |
| DENIS_0844 | pyruvate formate-lyase | *Ca*. Moduliflexus flocculans | 0 | 71.1 |
| DENIS_1896 | polysaccharide deacetylase | *Ca*. Moduliflexus flocculans | 1.2E-159 | 67.0 |
| DENIS_2093 | transposase | *Ca*. Moduliflexus flocculans | 2.89E-40 | 57.3 |
| DENIS_2422 | SsuB protein | *Ca*. Moduliflexus flocculans | 5.55E-66 | 49.1 |
| DENIS_2423 | SsuC protein | *Ca*. Moduliflexus flocculans | 3.31E-74 | 47.4 |
| DENIS_3040 | hypothetical protein U14_04437 | *Ca*. Moduliflexus flocculans | 2.44E-24 | 35.0 |
| DENIS_3119 | transposase | *Ca*. Moduliflexus flocculans | 1.54E-38 | 50.4 |
| DENIS_3264 | hypothetical protein U14_04635 | *Ca*. Moduliflexus flocculans | 3.44E-57 | 41.4 |
| DENIS_3407 | transcriptional regulator, LysR family | *Ca*. Moduliflexus flocculans | 7.9E-94 | 47.0 |
| DENIS_3774 | NUDIX hydrolase | *Ca*. Moduliflexus flocculans | 7.08E-92 | 57.3 |
| DENIS_4639 | hypothetical protein U14_04437 | *Ca*. Moduliflexus flocculans | 2.79E-26 | 35.2 |
| DENIS_4935 | hypothetical protein U14_00770 | *Ca*. Moduliflexus flocculans | 6.68E-45 | 51.1 |
| DENIS_0031 | hypothetical protein U27_01213 | *Ca*. Vecturithrix granuli | 8.92E-31 | 79.7 |
| DENIS_0129 | endonuclease/exonuclease/phosphatase family protein | *Ca*. Vecturithrix granuli | 1.35E-126 | 60.1 |
| DENIS_0162 | aminotransferase class I and II | *Ca*. Vecturithrix granuli | 1.23E-169 | 60.7 |
| DENIS_0290 | hypothetical protein U27_06677 | *Ca*. Vecturithrix granuli | 1.66E-141 | 38.8 |
| DENIS_0485 | MscS Mechanosensitive ion channel | *Ca*. Vecturithrix granuli | 0 | 41.3 |
| DENIS_1191 | putative amino acid ABC transporter | *Ca*. Vecturithrix granuli | 3.98E-80 | 56.3 |
| DENIS_1279 | putative transcriptional regulator, GntR family | *Ca*. Vecturithrix granuli | 0 | 68.9 |
| DENIS_1432 | probable amino acid ABC transporter, permease protein | *Ca*. Vecturithrix granuli | 3.86E-175 | 64.9 |
| DENIS_1433 | probable amino acid ABC transporter, permease protein | *Ca*. Vecturithrix granuli | 4.11E-170 | 56.6 |
| DENIS_1998 | two-component hybrid sensor and regulator | *Ca*. Vecturithrix granuli | 2.34E-133 | 56.0 |
| DENIS_1999 | multi-sensor hybrid histidine kinase | *Ca*. Vecturithrix granuli | 3.61E-145 | 42.6 |
| DENIS_2001 | putative two-component system sensor protein | *Ca*. Vecturithrix granuli | 0 | 43.6 |
| DENIS_2048 | transcriptional regulator, AraC family | *Ca*. Vecturithrix granuli | 1.39E-87 | 50.0 |
| DENIS_2049 | branched-chain amino acid permease (azaleucine resistance)-like protein | *Ca*. Vecturithrix granuli | 5.73E-116 | 69.5 |
| DENIS_2317 | hypothetical protein U27_05584 | *Ca*. Vecturithrix granuli | 8.45E-92 | 44.6 |
| DENIS_2405 | 3-dehydroquinate dehydratase | *Ca*. Vecturithrix granuli | 1.12E-80 | 75.0 |
| DENIS_2785 | ABC transporter permease protein | *Ca*. Vecturithrix granuli | 1.02E-157 | 70.0 |
| DENIS_2799 | hypothetical protein U27_04998 | *Ca*. Vecturithrix granuli | 5.67E-74 | 59.1 |
| DENIS_3278 | methyl-accepting chemotaxis sensory transducer | *Ca*. Vecturithrix granuli | 0 | 38.6 |
| DENIS_4012 | putative cyclohexadienyl dehydratase | *Ca*. Vecturithrix granuli | 1.18E-139 | 73.3 |
| DENIS_4056 | restriction endonuclease | *Ca*. Vecturithrix granuli | 3.32E-76 | 78.2 |
| DENIS_4220 | hypothetical protein U27_01123 | *Ca*. Vecturithrix granuli | 1.51E-09 | 28.7 |
| DENIS_4393 | response regulator receiver domain protein | *Ca*. Vecturithrix granuli | 1.94E-67 | 54.9 |
| DENIS_4396 | response regulator receiver domain protein | *Ca*. Vecturithrix granuli | 0 | 52.4 |
| DENIS_4398 | CheR methyltransferase, SAM binding domain protein | *Ca*. Vecturithrix granuli | 3.61E-114 | 44.4 |
| DENIS_4611 | hypothetical protein U27_01909 | *Ca*. Vecturithrix granuli | 4.65E-45 | 70.3 |
| DENIS_4612 | hypothetical protein U27_01908 | *Ca*. Vecturithrix granuli | 3.19E-64 | 65.4 |
| DENIS_4840 | HAD-superfamily hydrolase, subfamily IA, variant 3 | *Ca*. Vecturithrix granuli | 4.35E-55 | 42.2 |
| DENIS_4889 | lytic transglycosylase catalytic | *Ca*. Vecturithrix granuli | 1.42E-161 | 44.2 |
| DENIS_0195 | hypothetical protein CSB45_04955 | candidate division KSB3 bacterium | 1.11E-126 | 36.1 |
| DENIS_0274 | hypothetical protein CSA56_01490, partial | candidate division KSB3 bacterium | 3.71E-60 | 36.1 |
| DENIS_0839 | hypothetical protein CSA56_07410 | candidate division KSB3 bacterium | 5.65E-59 | 68.2 |
| DENIS_0840 | hypothetical protein CSA56_03965 | candidate division KSB3 bacterium | 1.82E-49 | 53.2 |
| DENIS_1762 | membrane dipeptidase | candidate division KSB3 bacterium | 1.19E-169 | 66.6 |
| DENIS_1942 | acetylesterase | candidate division KSB3 bacterium | 3.68E-167 | 68.9 |
| DENIS_2494 | succinate dehydrogenase/fumarate reductase iron-sulfur subunit | candidate division KSB3 bacterium | 3.25E-164 | 85.9 |
| DENIS_2758 | hypothetical protein CSA56_17280 | candidate division KSB3 bacterium | 0 | 50.5 |
| DENIS_2759 | hypothetical protein CSA56_17275 | candidate division KSB3 bacterium | 0 | 68.4 |
| DENIS_2761 | RNA-dependent DNA polymerase | candidate division KSB3 bacterium | 0 | 63.4 |
| DENIS_4067 | ABC transporter substrate-binding protein | candidate division KSB3 bacterium | 1.52E-115 | 44.9 |
