# Supplementary material for: Genomic Characteristics of Desulfonema ishimotonii Tokyo 01T Implying Horizontal Gene Transfer Among Phylogenetically Dispersed Filamentous Gliding Bacteria
Source: Front Microbiol. 2019 Feb 19;10:227. doi: 10.3389/fmicb.2019.00227 (PMC6390638; doi:10.3389/fmicb.2019.00227)
Supplement: Supplementary file 8 [file Table_5.DOCX]

**Table S5**. List of DNA repair genes in genome of strain Tokyo 01^T^. The column of organism indicates organisms which had the highest similarity with each of DNA repair enzyme. Genes present in two copies in the chromosome are marked in bold.

| Repair pathway | Gene name | locus_tag | Organism |
| --- | --- | --- | --- |
| Recombination repair | *recB* | DENIS_3009 | *Desulfobacteraceae bacterium* 4572_88 |
|  | *recC* | DENIS_2973 | *Desulfococcus multivorans* |
|  | *recD* | DENIS_3542 | *Desulfobacteraceae* bacterium IS3 |
|  | *recJ* | DENIS_2662 | *Desulfococcus multivorans* |
|  | *recN* | DENIS_3773 | *Desulfobacteraceae* bacterium 4572_88 |
|  | *recO* | DENIS_5135 | Deltaproteobacteria bacterium CG1_02_45_11 |
|  | ***recQ*** | DENIS_2396 DENIS_3915 | *Bathymodiolus septemdierum* thioautotrophic gill symbiont, *Ca.* Desantisbacteria bacterium CG2_30_40_21 |
|  | *recR* | DENIS_0260 | *Desulfobacteraceae* bacterium IS3 |
|  | *recA* | DENIS_3608 | *Desulfococcus multivorans* |
|  | *recG* | DENIS_5138 | *Desulfobacteraceae* bacterium 4572_88 |
|  | *ruvA* | DENIS_2483 DENIS_5175 | *Desulfobacterales* bacterium PC51MH44 *Desulfobacteraceae* bacterium IS3 |
|  | *ruvB* | DENIS_2480 | *Desulfobacteraceae* bacterium 4572_123 |
|  | *ruvC* | DENIS_2484 | *Desulfobacterales* bacterium S5133MH16 |
| Nucleotide excision repair  (NER) | *uvrA* | DENIS_1209 | *Desulfococcus multivorans* |
|  | ***uvrB*** | DENIS_1591 DENIS_1592 | *Desulfococcus multivorans* *Desulfobacteraceae* bacterium 4572_88 |
|  | *uvrC* | DENIS_1589 | *Desulfobacteraceae* bacterium 4572_88 |
|  | ***uvrD*** | DENIS_2690 DENIS_4485 | *Methanosarcina mazei, Desulfococcus multivorans* |
