# Supplementary material for: Genomic Characteristics of Desulfonema ishimotonii Tokyo 01T Implying Horizontal Gene Transfer Among Phylogenetically Dispersed Filamentous Gliding Bacteria
Source: Front Microbiol. 2019 Feb 19;10:227. doi: 10.3389/fmicb.2019.00227 (PMC6390638; doi:10.3389/fmicb.2019.00227)
Supplement: Supplementary file 9 [file Table_6.DOCX]

**Table S6**. List of genes for restriction-modification system in genome of strain Tokyo 01^T^. The column of taxon showed the phylogenetic affiliation of organism with the best BLASTP hit against each gene. Re, restriction enzyme; Mt, DNA methyltransferase.

| Taxon | Type I | | Type II | | Type III | | Type IV |
| --- | --- | --- | --- | --- | --- | --- | --- |
|  | Re | Mt | Re | Mt | Re | Mt | Re |
| *Deltaproteobacteria* | DENIS_2347 DENIS_1776 | DENIS_2351 DENIS_1771 |  | DENIS_2210 DENIS_3499 | DENIS_2926 | DENIS_2738 | DENIS_0095 DENIS_0096 |
| *Gammaproteobacteria* | DENIS_2349 | DENIS_1733 | DENIS_4449 DENIS_5128 DENIS_0311 | DENIS_2784 DENIS_1691 DENIS_3029 |  |  |  |
| *Betaproteobacteria* | DENIS_1735 |  |  |  |  |  |  |
| *Elusimicrobia* | DENIS_1734 |  |  |  |  |  |  |
| *Chloroflexi* |  |  | DENIS_3904 | DENIS_1339 |  |  |  |
| *Ignavibacteria* |  |  | DENIS_1400 | DENIS_3400 |  |  |  |
| *Firmicutes* | DENIS_1775 |  |  |  |  |  |  |
| *Ca.* NC10 |  |  |  |  | DENIS_2925 |  |  |
| *Alphaproteobacteria* |  |  | DENIS_3903 |  |  |  |  |
| *Archaea* |  |  |  | DENIS_4273 | DENIS_5141 | DENIS_5140 |  |
| *Cyanobacteria* |  |  | DENIS_3092 | DENIS_2587 DENIS_3231 |  |  |  |
| *Bacteroidetes* |  |  |  | DENIS_0778 |  |  | DENIS_4846 |
| *Balneoraeota* |  |  |  | DENIS_0536 |  |  |  |
| *Ca*. KSB |  |  |  | DENIS_3916 |  |  | DENIS_4056 |
| *Planctomycetes* |  |  |  | DENIS_4500 DENIS_5129 |  |  |  |
